# Genome-wide analysis of the diatom cell cycle unveils a novel type of cyclins involved in environmental signaling

**DOI:** 10.1186/gb-2010-11-2-r17

**Published:** 2010-02-08

**Authors:** Marie JJ Huysman, Cindy Martens, Klaas Vandepoele, Jeroen Gillard, Edda Rayko, Marc Heijde, Chris Bowler, Dirk Inzé, Yves Van de Peer, Lieven De Veylder, Wim Vyverman

**Affiliations:** 1 Protistology and Aquatic Ecology, Department of Biology, Ghent University, Krijgslaan 281-S8, 9000 Gent, Belgium; 2 Department of Plant Systems Biology, Flanders Institute for Biotechnology (VIB), Technologiepark 927, 9052 Gent, Belgium; 3 Department of Plant Biotechnology and Genetics, Ghent University, Technologiepark 927, 9052 Gent, Belgium; 4 Département de Biologie, Ecole Normale Supérieure, Centre National de la Recherche Scientifique, Unité Mixte de Recherche 8186, rue d'Ulm 45, 75230 Paris Cedex 05, France

## Abstract

**Background:**

Despite the enormous importance of diatoms in aquatic ecosystems and their broad industrial potential, little is known about their life cycle control. Diatoms typically inhabit rapidly changing and unstable environments, suggesting that cell cycle regulation in diatoms must have evolved to adequately integrate various environmental signals. The recent genome sequencing of *Thalassiosira pseudonana *and *Phaeodactylum tricornutum *allows us to explore the molecular conservation of cell cycle regulation in diatoms.

**Results:**

By profile-based annotation of cell cycle genes, counterparts of conserved as well as new regulators were identified in *T. pseudonana *and *P. tricornutum*. In particular, the cyclin gene family was found to be expanded extensively compared to that of other eukaryotes and a novel type of cyclins was discovered, the diatom-specific cyclins. We established a synchronization method for *P. tricornutum *that enabled assignment of the different annotated genes to specific cell cycle phase transitions. The diatom-specific cyclins are predominantly expressed at the G1-to-S transition and some respond to phosphate availability, hinting at a role in connecting cell division to environmental stimuli.

**Conclusion:**

The discovery of highly conserved and new cell cycle regulators suggests the evolution of unique control mechanisms for diatom cell division, probably contributing to their ability to adapt and survive under highly fluctuating environmental conditions.

## Abstract 2

Genes controlling the cell cycle in two diatoms have been identified and functionally characterized, revealing environmental regulation of the cell cycle.

## Background

Diatoms (Bacillariophyceae) are unicellular photosynthetic eukaryotes responsible for approximately 20% of the global carbon fixation [1,2]. They belong to the Stramenopile algae (chromists) that most probably arose from a secondary endosymbiotic process in which a red eukaryotic alga was engulfed by a heterotrophic eukaryotic host approximately 1.3 billion years ago [3,4]. This event led to an unusual combination of conserved features with novel metabolism and regulatory elements, as recently confirmed by whole-genome analysis of *Thalassiosira pseudonana *and *Phaeodactylum tricornutum *[5-7], which are representatives of the two major architectural diatom types, the centrics and the pennates, respectively.

Besides their huge ecological importance, diatoms are interesting from a biotechnological perspective as producers of a variety of metabolites (including oils, fatty acids, and pigments) [8,9], and because of their highly structured mesoporous cell wall, made of amorphous silica [10]. Thus, understanding the basic mechanisms controlling the diatom life cycle will be important to comprehend their ecological success in aquatic ecosystems and to control and optimize diatom growth for commercial applications.

As predominant organisms in marine and freshwater ecosystems, diatoms often encounter rapid and intense environmental fluctuations (for example, light and nutrient supply) [11] that might have dramatic effects on cell physiology and viability. Therefore, cell cycle regulation in diatoms most probably involves efficient signalling of different environmental cues [12]. Recent studies illustrate how diatoms can acclimate rapidly to iron limitation [13,14] and phosphorus scarcity [15] through biochemical reconfiguration or maintenance of internal reservoirs and how their cell fate can be determined by perception of diatom-derived reactive aldehydes [16,17]. Furthermore, in *P. tricornutum*, a new blue light sensor (cryptochrome/photolyase family member 1) has been discovered with dual activity as a 6-4 photolyase and a blue-light-dependent transcription regulator [18]. Thus, diatoms are expected to possess complex fine-tuned signalling networks that integrate diverse stimuli with the cell cycle. The recent availability of genome data of *T. pseudonana *[5] and *P. tricornutum *[6] now provides the basis to explore how the cell cycle machinery has evolved in diatoms.

Efficient molecular regulation of the cell cycle is crucial to ensure that structural rearrangements during cell division are coordinated and that the genetic material is replicated and distributed correctly. In eukaryotes, the mitotic cell cycle comprises successive rounds of DNA synthesis (S phase) and cell division (mitosis or M phase) separated from each other by two gap (G1 and G2) phases [19]. Passage through the different cell cycle phases is controlled at multiple checkpoints by an evolutionarily conserved set of proteins, the cyclin-dependent kinases (CDKs) and cyclins (reviewed in [19,20]). Together, these proteins can form functional complexes, in which the CDKs and cyclins act as catalytic and regulatory subunits, respectively. Various types of CDKs and cyclins exist and they generally regulate the cell cycle, but some can be involved in other processes, such as transcriptional control or splicing [21,22].

In eukaryotes, activity of CDK-cyclin complexes is mainly controlled by (de)phosphorylation of the CDK subunits and interaction with inhibitors or scaffolding proteins [23]. Regulators include CDK-activating kinases (CAKs) [24,25], members of the WEE1/MYT1/MIK1 kinase family and CDC25 phosphatases that carry out inhibitory phosphorylation and dephosphorylation [26], as well as CDK inhibitors (CKIs) [23] and the scaffolding protein CKS1/Suc1 [27,28].

The aim of this work was to reveal the molecular network of cell cycle regulators in *P. tricornutum*, a species used for decades as a model diatom for physiological studies. *P. tricornutum *is a coastal diatom, typically found in highly unstable environments, and its cells can easily acclimate to environmental changes [13,29]. Key cell cycle regulators (CDKs, CDK interactors, and cyclins) were annotated and their transcript expression profiled during synchronized growth in *P. tricornutum*. The results indicate that diatom cell division is controlled by a combination of conserved molecules found in yeast, animals and/or plants, and novel components, including diatom-specific cyclins that probably transduce the environmental status of the cells to the cell cycle machinery.

## Results and discussion

### Annotation of the cell cycle genes in diatoms

The following cell cycle gene families were selected for comprehensive analysis: CDKs, cyclins, CKS1/suc1, WEE1/MYT1/MIK1, CDC25, and CKIs. These gene families were annotated functionally on the basis of their homology with known cell cycle genes in other organisms (see Materials and methods). The results of this family-wise annotation are discussed below and summarized in Table 1 and Additional file 1. The nomenclature of all identified proteins is according to that used in other protists for which cell cycle gene annotation was available [30,31].

**Table 1:** Overview and evolutionary conservation of the different core cell cycle gene families

	Number of copies				
Cell cycle gene	Phatr^a^	Arath^a, b^	Ostta^a, c^	Sacce^a, c^	Homsa^a, c^
*CDKA*	2^d^	1	1	1	3
*CDKB*	-	4	1	-	-
*CDKC*	2	2	1	1	1
*CDKD*	1	3	1	-	1
*CDKF*	-	1	-	1	1
*CYCA*	1?	10	1	NA	NA
*CYCB*	2?	9	1	NA	NA
*CYCD*	1?	10	1	NA	NA
*CYCH*	1?	1	1	NA	NA
*CDC25*	-	-	1^e^	1	3
*Wee1/Myt1/Mik1*	1	1	2	2	2
*CKS*	1	2	1	1	2
*CKI*	-	7	1	1	8

^a^Abbreviations: Phatr, *Phaeodactylum tricornutum*; Arath, *Arabidopsis thaliana*; Ostta, *Ostreococcus tauri*; Sacce, *Saccharomyces cerevisiae*; Homsa, *Homo sapiens*. ^b^Data taken from [67]. ^c^Data taken from [30].^d^One of these genes shows some *CDKB *characteristics. ^e^Classification uncertain because of weak phylogeny. NA, not available due to other classification nomenclature.

### Cell cycle synchronization and expression analysis

**Figure 1:**
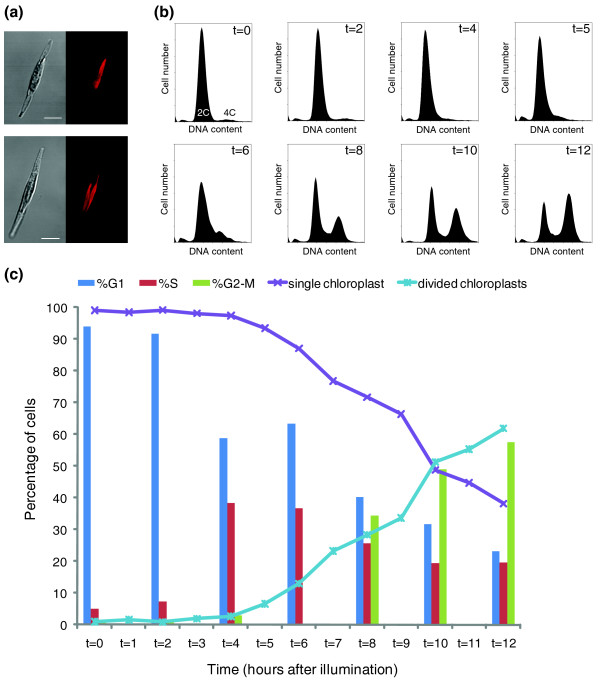
**Synchronization of the cell cycle in *P. tricornutum***. **(a) **Confocal images of a dark-arrested cell (upper panel) showing a single parietal chloroplast and a cell after 12 h illumination (lower panel) showing divided and translocated daughter chloroplasts. Red, autofluorescence of the chloroplast. Scale bar: 5 μm. **(b) **Validation of synchronization of the cell cycle of *P. tricornutum *by flow cytometry. DNA content (abscissa) is plotted against cell number (ordinate). After a 20-h dark period, most of the cells are blocked in G1 phase (t = 0 to 4 h), indicated by the single 2C peak. After reillumination, cells proceed synchronously with their cell cycle, going through S phase (between t = 4 and 7 h), visible as the broadening and lowering of the 2C peak, and G2-M phase (t = 8 to 12 h), indicated by the accumulation of 4C cells. **(c) **Histogram indicating the proportion of cells in a certain cell cycle phase and chloroplast conformation during the cell cycle. Divided chloroplasts were observed starting from 5 h after illumination, after S-phase onset.

To validate the predicted functions of the annotated genes, we examined their transcript expression during the cell cycle. To synchronize cell division in *P. tricornutum*, we subjected exponentially growing cells to a prolonged dark period, which arrests the cells in the G1 phase [32] (Figure 1; Additional file 2), and released the cells synchronously from this arrest point by illumination. A comparable method had been applied successfully to synchronize growth in a closely related diatom, *Seminavis robusta *[33]. Microscopic observations of the dark-arrested *P. tricornutum *cultures showed that all cells contained a single undivided chloroplast (Figure 1a, upper panel). Accordingly, in flow cytometric histograms, the dark-arrested cells showed only a 2C peak (Figure 1b and Additional file 2, t = 0), confirming the G1 phase identity of cells containing a single chloroplast. When cells were released from the dark arrest, the population of bi-chloroplastidic cells steadily increased and cells entered the S phase, as observed by flow cytometry (Additional file 2, upper panel). However, the level of synchrony decreased at later time points (from 10 h after the dark release onward), probably because cells entered the next cell division cycle at the moment other cells still had to pass through M phase (Additional file 2). To circumvent this problem and to obtain an enrichment of cells in M phase during the later time points (Additional file 2), the metaphase blocker nocodazole was added at the time of re-illumination [34], but without major effect on cell cycle progression (Additional file 2).

To monitor gene expression during the different cell cycle phases, exponentially growing cells were synchronized in the presence of nocodazole (Figure 1b, c). Automated analysis of the flow histograms indicated that G1-phase cells were dominant during the first 4 h of re-illumination; from 4 to 7 h, cells went through S phase, as seen by the broadening and lowering of the 2C peak, while cells went mainly through the G2 and M phases at 8 to 12 h (Figure 1b, c). In *S. robusta*, chloroplast division had been found to take place only after S-phase onset [33]. Chloroplast division in *P. tricornutum *was observed starting from 5 h after illumination, confirming the S-phase timing determined by flow cytometry (Figure 1a, lower panel, and 1c). The duration of the cell cycle after the synchronization procedure was comparable with that of cultures grown under standard conditions (approximately one division per day; Additional file 3). For downstream analysis, at hourly intervals after illumination, samples were taken for expression analysis by real-time quantitative polymerase chain reaction (qPCR).

### CDKs and CDK interactors

#### CDKs

CDKs are serine/threonine kinases that play a central role in cell cycle regulation and other processes, such as transcriptional control. Yeast uses only one single PSTAIRE-containing CDK for cell cycle progression [35,36], while higher organisms encode different CDKs implicated in cell division. The most conserved CDKs contain a PSTAIRE cyclin-binding motif [19,20]. In plants, the PSTAIRE-containing CDK had been designated CDKA and is active during both G1-to-S and G2-to-M transitions [19]. The plant-specific B-type CDKs contain a P [P/S]T [A/T]LRE motif and are active during the G2 and M phases [19]. In animals, three PSTAIRE (Cdk1, Cdk2, and Cdk3) and two P(I/L)ST(V/I)RE (Cdk4 and Cdk6) CDKs are involved in cell cycle control, although evidence has been found recently that only Cdk1 is really required to drive cell division [20,37].

**Figure 2:**
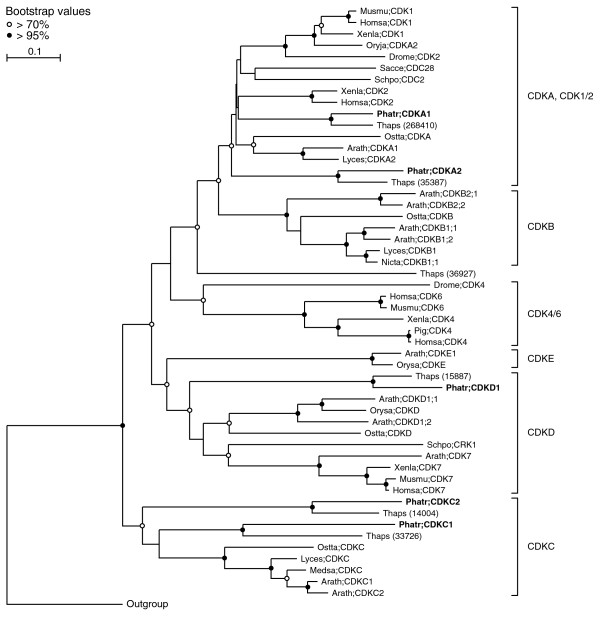
**Phylogenetic analysis of the cyclin-dependent kinases of *P. tricornutum***. Neighbor-joining tree (TREECON, Poisson correction, 1,000 replicates) of the CDK family. The *P. tricornutum *sequences are shown in bold. Abbreviations: Arath, *Arabidopsis thaliana*; Drome, *Drosophila melanogaster*; Homsa, *Homo sapiens*; Lyces, *Lycopersicon esculentum*; Medsa, *Medicago sativa*; Musmu, *Mus musculus*; Nicta, *Nicotiana tabacum*; Oryja, *Oryza japonica*; Orysa, *Oryza sativa*; Ostta, *Ostreococcus tauri*; Phatr, *Phaeodactylum tricornutum*; Sacce, *Saccharomyces cerevisiae*; Schpo, *Schizosaccharomyces pombe*; Thaps, *Thalassiosira pseudonana*; and Xenla, *Xenopus laevis*.

**Figure 3:**
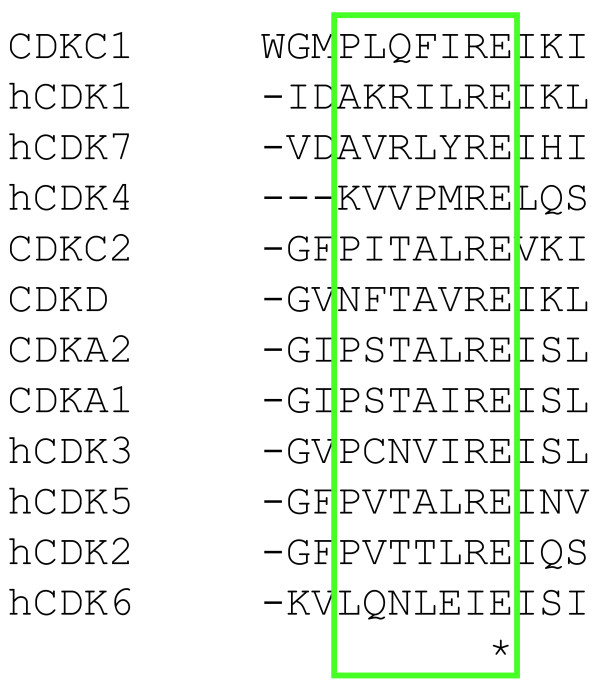
**Cyclin-dependent kinase cyclin-binding motifs**. Alignment of the cyclin-binding motifs of all annotated CDKs in *P. tricornutum*. The motifs are indicated in the green box. Conserved residues are marked by an asterix in the bottom line.

**Figure 4:**
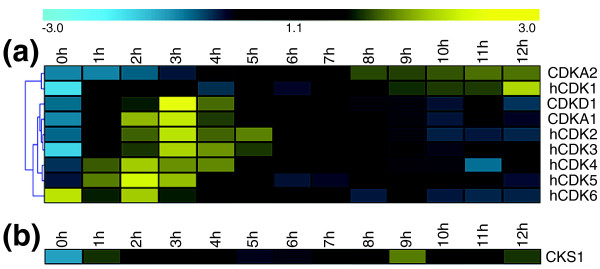
**Hierarchical average linkage clustering of the expression profiles of cyclin-dependent kinases and their interactors in *P. tricornutum***. **(a) **Members of the CDK family. **(b) **CKS1. h, hypothetical.

Five CDKs could be identified in *P. tricornutum *(Table 1), of which two clustered together with the CDKA (plant)/CDK1-2 (animal) family in the phylogenetic tree (Figure 2). CDKA1 contains the typical PSTAIRE cyclin-binding motif (Figure 3) and its mRNA levels were high during late G1 and S phase (Figure 4a), suggesting a role at the G1-to-S transition. CDKA2 shows a PSTALRE motif (Figure 3), which is a midway motif between the CDKA hallmark PSTAIRE and the plant-specific CDKB hallmark P [P/S]T [A/T]LRE. The mRNA levels of CDKA2 were elevated in G2/M cells (Figure 4a). No homologs of the metazoan CDK4/6 family were found in *P. tricornutum*.

CDKC, CDKD and CDKE (designated Cdk9, Cdk7 and Cdk8 in animals, respectively) are kinases related to CDKA [38]. C-type CDKs (CDKC and Cdk9) and Cdk8 have been shown to associate with transcription initiation complexes and, thus, to play a role in transcriptional control [39,40]. Additionally, CDKC2 is active in spliceosomal dynamics in plants [22] and CDKE controls floral cell differentiation [41]. We identified two C-type CDKs (Table 1), CDKC1 and CDKC2 (Figure 2a) with PITALRE and PLQFIRE cyclin-binding motifs, respectively (Figure 3). No CDKE homolog was found in *P. tricornutum*. Both *CDKC *genes had relatively low mRNA levels throughout the cell cycle without any discernible cell cycle phase pattern (data not shown). Thus, like in other eukaryotes, *CDKC *expression probably does not depend on the cell cycle phase in *P. tricornutum*, but it might be involved in other processes, such as transcription or splicing. One CDKD was identified (*CDKD1*) in *P. tricornutum *(Table 1 and Figure 2a). D-type CDKs are known to interact with H-type cyclins to form a CAK complex [24]. We found that *CDKD1 *mRNA levels were high at the G1-to-S phase transition (Figure 4a). Another CDK variant, CDKF, has only been found in plants, where it functions as a CAK-activating kinase (CAKAK) [24]. No members of the CDKF family were identified in *P. tricornutum*, confirming that the CAKAK pathway is specific to plants and should have evolved within the green lineage (Table 1).

In addition, we identified seven hypothetical CDKs (hCDKs; Additional file 1) with divergent cyclin-binding domains (Figure 3) that could not be integrated into the phylogenetic tree due to high sequence divergence. The expression levels of several of these hCDKs were modulated during the cell cycle (Figure 4a). The *hCDK1 *mRNA levels were the highest during G2-M, whereas those of *hCDK6 *were up-regulated during G1 phase and *hCDK2*, *hCDK3*, *hCDK4*, and *hCDK5 *were predominantly expressed at G1 and/or S phase. For *hCDK7*, no reproducible expression pattern was found (data not shown).

#### CDK subunit

CDK subunit (CKS) proteins act as docking factors that mediate the interaction of CDKs with putative substrates and regulatory proteins [27]. In *P. tricornutum*, one *CKS *gene was found (*CKS1*; Table 1) of which the mRNA levels were mainly high in G2/M cells (Figure 4b).

#### WEE1/MYT1/MIK1 kinases

WEE1/MYT1/MIK1 kinases inhibit cell cycle progression through phosphorylation of CDKs [26]. In yeast and animals, MYT1 is a membrane-associated kinase that phosphorylates Thr14 of Cdc2 proteins, as well as Tyr15, which is also a target of WEE1, a nucleus-localized kinase [42,43]. A single CKI could be identified in *P. tricornutum*, belonging to the MYT1 family (Table 1; Additional file 4) [42]. In *Arabidopsis thaliana*, the inhibitory kinase corresponds to WEE1 [44], while the green alga *Ostreococcus tauri *expresses both *WEE1 *[30] and *MYT1 *(unpublished data), like animals do [42] (Table 1). Expression of the *P. tricornutum MYT1 *kinase was not associated with a specific cell cycle phase (data not shown). Because *MYT1 *is probably implicated in stress responses during the cell cycle [45], it is possible that the imposed dark arrest or addition of nocodazole influenced the mRNA levels of *MYT1*, with too much variability in its expression profile as a consequence.

#### CDC25 phosphatase

As antagonists of the WEE1/MYT1/MIK1 kinases, CDC25 phosphatases activate CDKs [26]. In contrast to the presence of a counteracting kinase, no CDC25 phosphatase could be identified in *P. tricornutum *(Table 1) or in *T. pseudonana*. Both *Arabidopsis *and *Oryza sativa *also lack a functional CDC25 [46,47] and, in plants, CDC25-mediated regulatory mechanisms have been proposed to be replaced by a mechanism governed by the plant-specific B-type CDKs [48]. In *P. tricornutum*, no true B-type CDK homolog could be found, but CDKA2, classified by weak homology as A-type CDK class, possessed a PSTALRE cyclin-binding motif (Figure 3), which is halfway between the CDKA and CDKB hallmarks. This motif also occurred in the *Dictyostelium discoideum *CDC2 homolog [49] and in the *O. tauri *CDKB protein [30]. The PSTALRE motif is present as well in the CDKA2 homolog of *T. pseudonana *(Thaps3_35387; Figure 2a), confirming that this subtype could generally be found in diatoms. Moreover, *CDKA2 *was expressed during G2-M (Figure 4a), the expected time of action of a B-type CDK. Although further in-depth biochemical research will be required to determine its true physiological function, the presence of this A/B-type CDK might explain the absence of a CDC25 phosphatase in diatoms. Alternatively, if the sequence of the CDC25 phosphatase had diverged to such an extent in diatoms, it might be not detectable by sequence homology, as already suggested for higher plants as well [50].

#### CDK inhibitors

CDK-cyclin complexes can be inactivated by CKIs, including the members of the INK4 family and the Cip/Kip family in animals [51], or Kip-related proteins and SIAMESE proteins in plants [52,53]. CKIs are mainly low-molecular-weight proteins that inhibit CDK activity by tight association in response to developmental or environmental stimuli [23,51,54]. Despite extensive sequence similarity searches for CKIs, no homologs could be identified in *P. tricornutum*, which is not so surprising given the high sequence diversity of this cell cycle family [52]. These inhibitory proteins are most probably present in *P. tricornutum*, but their identification will require more advanced molecular techniques.

### Cyclins

#### The cyclin gene family is expanded in diatoms

**Figure 5:**
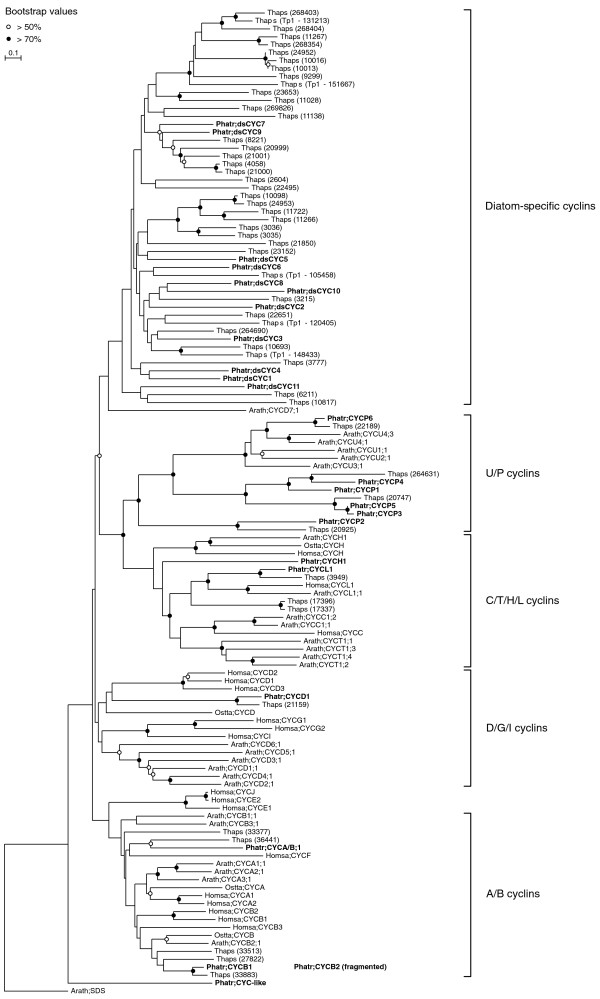
**Phylogenetic analysis of the cyclins of *P. tricornutum***. Neighbor-joining tree (TREECON, Poisson correction, 500 replicates) of the cyclin family. The *P. tricornutum *sequences are shown in bold. Abbreviations: Arath, *Arabidopsis thaliana*; Homsa, *Homo sapiens*; Ostta, *Ostreococcus tauri*; Phatr, *Phaeodactylum tricornutum*; and Thaps, *Thalassiosira pseudonana*.

We found a large number of highly diverged cyclin genes in diatoms, of which 24 are in *P. tricornutum *(Additional file 1). Due to their high divergence, indicated by the low bootstrap values in the phylogenetic tree, the classification into different subclasses was not clear (Figure 5), as it was for the 52 putative cyclins identified in *T. pseudonana *[55]. Moreover, many represent a novel class of cyclins, which we designated diatom-specific cyclins (dsCYCs).

To investigate whether the expansion of the cyclin gene family is specific to diatoms, we compared cyclin abundance among a representative set of Chromalveolates (Stramenopiles, Apicomplexa, and Ciliates; Table 2) for which genome data are available [56-64] and have been pre-processed in a previous study [65]. Because of the lack of cell cycle gene annotation in all investigated species, we first screened for cyclin genes, which allowed us to create a reference dataset for analyzing cyclin evolution. We searched the different genomes for proteins that showed similarity to our cyclin HMMER profile and determined the number of proteins that contained an InterPro cyclin domain (Table 2). Generally, both detection methods yielded comparable results within all species (Table 2). An indication of the putative subclasses and function of the detected proteins is given by specific cyclin InterPro domains (Table 2). The proportion of the detected cyclin proteins relative to the predicted total gene number of each species revealed that, in the diatom genomes, cyclins are overrepresented compared to all investigated species, except for both *Cryptosporidium *species [57,58] and *Paramecium tetraurelia *[64] (Table 2). However, the total number of cyclins found in *Cryptosporidium *(12) is low compared to that in diatoms (28 in *P. tricornutum *and 57 in *T. pseudonana*). *Cryptosporidium *species are protozoan pathogens that depend on their hosts for nutrients. Moreover, Gene Ontology distribution for *Cryptosporidium *and *Plasmodium *is similar, indicating that no functional specialization of conserved gene families has occurred [58]. In *Paramecium tetrauleria*, the cyclin family is expanded as well. However, this species has a complex genome structure, possessing silent diploid micronuclei and polyploid macronuclei. Furthermore, *P. tetraurelia *underwent at least three whole-genome duplications, resulting in an apparent expansion of almost every gene family [64].

**Table 2:** Expansion of cyclin gene family in different representatives of the Chromalveolata

	Stramenopiles				Apicomplexa						Ciliates	
			
	Diatoms		Oomycetes									
										
	Phatr	Thaps	Phyra	Physo	Cryho	Crypa	Plafa	Playo	Thean	Thepa	Parte	Tetth
**General**												
Number of proteins matching the cyclin HMMER profile	28	57	19	19	12	12	5	5	8	8	144	29
Number of proteins with an InterPro cyclin domain	27	55	18	18	12	12	5	5	4	6	140	27
												
**Specific InterPro domains**												
IPR004367 Cyclin, C-terminal	7	9	4	5	-	-	-	-	-	-	19	6
IPR006670 Cyclin	6	18	7	7	2	2	2	1	2	1	94	20
IPR006671 Cyclin, N-terminal	18	45	9	10	1	1	-	-	-	-	96	20
IPR011028 Cyclin-like	27	55	18	18	11	11	5	5	4	6	140	27
IPR013763 Cyclin-related	21	47	13	13	6	6	3	2	2	2	72	22
IPR013922 Cyclin-related 2	1	1	1	1	3	3	1	1	1	1	21	1
IPR014400 Cyclin, A/B/D/E	2	4	2	3	1	1	-	-	-	-	42	3
IPR015429 Transcription regulator cyclin	4	3	6	5	2	2	2	2	2	2	6	3
IPR015432 Cyclin H	1	-	1	1	1	1	1	1	1	1	-	-
IPR015451 Cyclin D	-	4	-	-	1	-	-	-	-	-	-	-
IPR015452 G2/mitotic-specific cyclin B3	-	-	-	-	-	-	-	-	-	-	3	1
IPR015453 G2/mitotic-specific cyclin A	1	1	3	3	-	-	-	-	-	-	2	-
IPR015454 G2/mitotic-specific cyclin B	1	-	-	-	-	-	-	-	-	-	9	1
IPR017060 Cyclin L	-	-	1	1	-	-	1	1	-	-	2	-
												
**Total number of genes**	10,402	11,776	15,743	19,027	3,994	3,952	5,268	5,268	3,792	4,035	39,642	27,000
**Genome size (Mbp)**	27.4	32.4	65	95	9.16	9.11	22.85	23.1	8.35	8.3		104
**Cyclins/genes total (%)^a^**	0.27	0.48	0.12	0.10	0.30	0.30	0.09	0.09	0.21	0.20	0.36	0.11
**Cyclins/genes total (%)^b^**	0.26	0.47	0.11	0.09	0.30	0.30	0.09	0.09	0.11	0.15	0.35	0.10

Abbreviations: Phatr, *Phaeodactylum tricornutum*; Thaps, *Thalassiosira pseudonana*; Phyra, *Phytophthora ramorum*; Physo, *Phytophthora sojae*; Cryho, *Cryptosporidium hominis*; Crypa, *Cryptosporidium parvum*; Plafa, *Plasmodium falciparum*; Playo, *Plasmodium yoelii yoelii*; Thean, *Theileria annulata*; Thepa, *Theileria parva*; Parte, *Paramecium tetraurelia*, Tetth, *Tetrahymena thermophila*. ^a^Number of cyclins versus total number of genes calculated with the number of proteins that match our cyclin HMMER profile. ^b^Number of cyclins versus total number of genes calculated with the number of proteins with a InterPro cyclin domain.

In conclusion, the large number of cyclin genes in both diatoms does not seem to be shared with its closest related species, indicating that diatom cyclins could have evolved separately to acquire new specific functions. Although the cyclin family has been found to be expanded in both diatoms, the size of the cyclin gene family in *T. pseudonana *is larger than that in *P. tricornutum*, which seems to result mainly from the presence of a larger number of diatom-specific cyclins in *T. pseudonana *(Figure 5). The biological cause of the changes in the cyclin family size remains unknown, although natural selection due to differential habitats might have played a role, or alternatively, random gene loss or gain might have occurred over long time stretches, as both species diverged at least 90 million years ago [6]. Genome sequence data of other diatom species are currently being generated (for example, for *Fragilariopsis cyclindrus *and *Pseudo-nitzschia multiseries*) and will help to shed light on cyclin gene family evolution in diatoms.

#### Conserved cyclins

**Figure 6:**
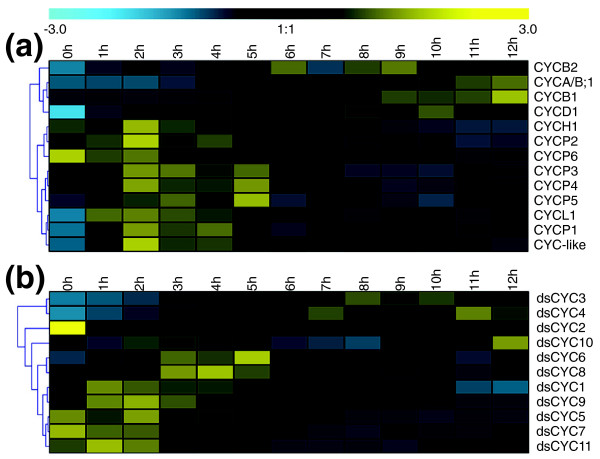
**Hierarchical average linkage clustering of the expression profiles of cyclin genes in *P. tricornutum***. **(a) **Cyclin genes. **(b) **dsCYCs. ds, diatom specific.

Cyclins can be functionally classified into two major groups: the cell cycle regulators and the transcription regulators. Generally, during the cell cycle, specific cyclins are associated with G1 phase (cyclin D), S phase (cyclins A and E), and mitosis (cyclins A and B) [66]. In *P. tricornutum*, we identified a single A/B-type cyclin gene (*CYCA/B;1*; Figure 5), which gradually accumulated its mRNA transcript during the G2 and M phases (Figure 6a). Both B-type cyclin genes (encoded by *CYCB1 *and *CYCB2*) (Figure 5) were predominately expressed in G2/M cells, but mRNA levels of *CYCB2 *accumulated earlier than those of *CYCB1 *(Figure 6a). The single D-type cyclin (encoded by *CYCD1*; Figure 2b) was mainly expressed during S and G2/M phase progression (Figure 6a). As in plants, CYCE seems to be absent in diatoms [67].

Cyclins with a regulatory role during transcription include those belonging to the classes C, H, K, L, and T [39]. However, some cyclins involved in transcriptional control might also have a function in cell cycle regulation. For example, besides being a transcriptional regulator, the human C-type cyclin is also involved in the control of cell cycle transitions [68] and H-type cyclins can regulate the cell cycle through interaction with D-type CDKs, thereby forming a CAK complex [24,69,70]. The latter is probably also true for the P. tricornutum CYCH1 (Figure 5) because it was coexpressed with CDKD1 during the cell cycle (Figure 6a). The single L-type cyclin (encoded by CYCL1; Figure 5) showed elevated mRNA levels at G1 and during S phase (Figure 6a). In animals, cyclin L (also called Ania-6) has previously been demonstrated to be an immediate early gene that could be involved in cell cycle re-entry [71,72].

Six cyclins in *P. tricornutum *clustered together with P-type cyclins (PHO80-like proteins, also called U-type cyclins; Additional file 1 and Figure 5) that are believed to play a role in phosphate signalling [73,74]. The mRNA levels of all P-type cyclin genes (*CYCP1*, *CYCP2*, *CYCP3*, *CYCP4*, *CYCP5*, and *CYCP6*) were high early during the time series (Figure 6a). One cyclin gene did not cluster with any of the represented classes and was annotated as CYC-like (Figure 5). The mRNA levels of this gene peaked during the G1 and S phases (Figure 6a).

#### Most diatom-specific cyclins are expressed early during the cell cycle

Eleven cyclin genes were identified that clustered only with cyclins of *T. pseudonana *(Figure 5). Therefore, we assigned these as dsCYC genes. *dsCYC3 *and *dsCYC4 *showed both high expression at the G2/M phases (Figure 6b). The mRNA levels of *dsCYC10 *were slightly up-regulated at the G1-to-S transition and reached a peak late during the cell cycle (Figure 6b). As the other *dsCYC *genes displayed increased mRNA levels during the G1 and/or S phases (*dsCYC1*, *dsCYC2*, *dsCYC5*, *dsCYC6*, *dsCYC7*, *dsCYC8*, *dsCYC9*, and *dsCYC11*; Figure 6b), some might function as immediate early genes controlled by light or mitogens.

Organisms living in aquatic environments, particularly in coastal regions, often have to cope with rapid and broad fluctuations in light intensity, temperature, nutrient availability, oxygen level, and salinity, all of which can have profound consequences on cell cycle progression. Comparative genome analyses of marine phytoplankton have revealed that coastal organisms contain genetic imprints indicative of adaptation to life under variable conditions [75,76], including distinct proteins coding for photosynthesis and light harvesting, additional two-component regulatory systems, novel carbon-concentrating mechanisms, transcription of transporters and assimilation proteins for the uptake of alternative nitrogen sources, and numerous metal transporter families and metal enzymes [75,76]. Similar adaptation imprints were also found in the diatom genomes [5,6]. Nevertheless, because diatoms generally dominate the microplankton in temperate waters and coastal upwelling regions under favorable conditions [77], we expect diatoms to possess additional sophisticated fine-tuning systems enabling them to adjust the pace of the cell division rate in tune with the prevailing conditions.

Although in plants numerous copies of D-type cyclins integrate both external and internal signals into the cell cycle [19], in *P. tricornutum *only one *CYCD *was identified that was highly expressed late during the cell cycle (Figure 6a). Therefore, in diatoms CYCD probably does not play its classical role of G1-phase signal integrator, but might have acquired an alternative function in the G2-to-M transition as previously proposed for some D-type cyclins in plants [78]. On the other hand, the wide variety of *dsCYC *genes in diatoms expressed early during the cell cycle renders them plausible candidates to fulfil the task of signal integrators. Moreover, diatom-specific genes have been found to evolve faster than other genes in diatom genomes [6], indicating that these cyclin genes might have acquired novel and/or species-specific functions. Interestingly, other gene families expanded in diatoms include histidine kinases and heat shock factors, which are supposed to be involved in environmental sensing and expressed under certain growth conditions [6]. Thus, gene family expansion in diatoms could possibly be linked to the development of specific signal responses and adaptations to the environment.

### dsCYCs respond to nutrient availability

To investigate the role of the *dsCYC *genes during the cell cycle, we analyzed them in more detail. More specifically, we examined whether their transcription is affected by nutrient deprivation. Analysis of recently published expressed sequence tag data [79,80] illustrates the differential expression of *dsCYC3*, *dsCYC7*, and *dsCYC10 *across a range of environmental conditions (for example, nitrate-starved, nitrate-repleted, and iron-limited cultures). Moreover, a microarray analysis revealed that *dsCYC9 *transcript levels were higher in cultures grown in the presence of silica than those without silica [81].

**Figure 7:**
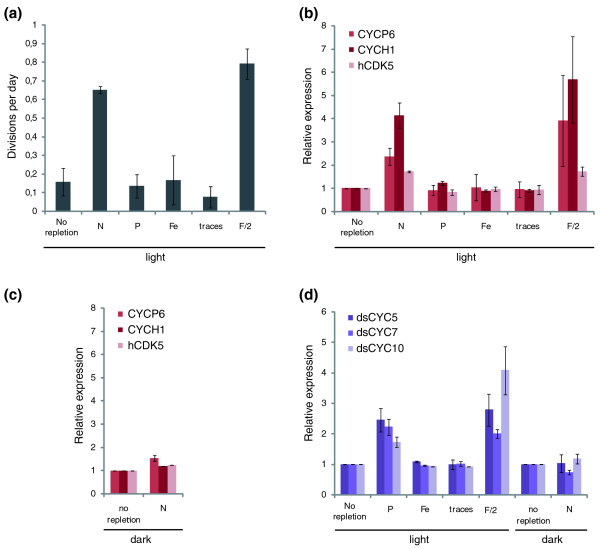
**Nutrient response of diatom-specific cyclins**. **(a) **Growth rate of different subcultures after repletion based on average cell density measurements at the time of and 3 days after repletion. These data indicate the ability of the cells to recover from starvation. **(b) **Expression profiles of early cell cycle genes at the time of sampling during the light experiment. **(c) **Expression profiles of early cell cycle genes at the time of sampling during the dark experiment. **(d) **dsCYCs responding to phosphate addition. Error bars represent standard errors of the mean of two biological replicates.

To examine whether *dsCYC *expression could be responsive to nutrient status during the cell cycle, we monitored mRNA levels in parallel with cell growth during nutrient starvation-repletion experiments. Exponentially growing cultures were nutrient-starved for 24 h and re-supplied with only nitrate, phosphate, iron, trace metals, the combination of all nutrients (positive control), or no nutrients (negative control). Three hours after nutrient supply, samples were collected for expression analysis. After nitrate repletion, cells reinitiated cell division at almost comparable levels to the positive control cultures, whereas repletion with phosphate, iron, or trace elements did not differ from the negative control (Figure 7a), indicating that nitrate is a cell cycle rate-limiting nutrient in *P. tricornutum*, as reported for other diatom species [82,83]. Nitrogen starvation in diatoms generally leads to a G1-phase arrest [82,83]. Thus, increased mRNA levels of early cell cycle-regulated genes are to be expected at the time of cell cycle reinitiation after nitrate repletion. Accordingly, early cell cycle genes (*CYCP6*, *CYCH1*, and *hCDK5*) were induced in the nitrate replete and positive control cultures (Figure 7b). To exclude cell cycle effects during sampling, the starvation experiment was repeated for nitrate repletion, but after imposing a 24-h dark arrest after starvation and re-supply of nitrate in complete darkness. In these cultures, the expression of the early cell cycle genes did not differ from that of the negative control after nitrate supply (Figure 7c), confirming that expression of *CYCP6*, *CYCH1*, and *hCDK5 *is linked to cell cycle re-entry rather than to the nitrate status of the cells.

In contrast to nitrate, cells resupplied with phosphate remained arrested (Figure 7a, b). Upon addition of phosphate, mRNA levels of *dsCYC5*, *dsCYC7 *and *dsCYC10 *were significantly higher than those of the negative control (Figure 7d), strongly suggesting that these genes might function as direct cell cycle signal integrators upon increase of phosphate levels. Upon replenishment with nitrate (in the dark), iron or trace elements, no effects on *dsCYC *gene expression were observed (Figure 7d and data not shown).

Nitrogen, together with the micronutrient iron, is generally considered to be a major limiting factor of primary production in the oceans [84]. Phosphate limitation, on the other hand, is considered to be less common, although it has been reported in certain oceanic areas [85] and has been hypothized recently to have been more wide-spread during the glacial periods [86]. As an important constituent of adenosine triphosphate, nucleic acids, and phospholipids, phosphorus is an important molecule not only for growth, but for almost all metabolic activities. Recently, diatoms have been shown to reduce their phosphorus demand upon phosphorus limitation, and to maintain growth by substituting phospholipids with non-phosphorus membrane lipids, only when nitrogen is not limiting [15].

In summary, these results reveal that some dsCYCs might be involved in environmental cell cycle control, functioning as nutrient signal integrators. All phosphate-responding *dsCYC *genes were expressed early during the synchronized time series (Figure 6b), fitting with a function in linking nutritional status and cell division start.

### Cell cycle biomarkers

The identification of the complete set of major cell cycle regulators in P. tricornutum, along with the determination of their temporal expression patterns, generates a basis for studying different cell cycle-related processes in diatoms. Diatom cell cycle biomarkers could be used to observe cell cycle effects in laboratory experiments, but they could also be highly valuable to monitor diatom life cycle events in the natural habitat, like bloom or rest periods.

**Figure 8:**
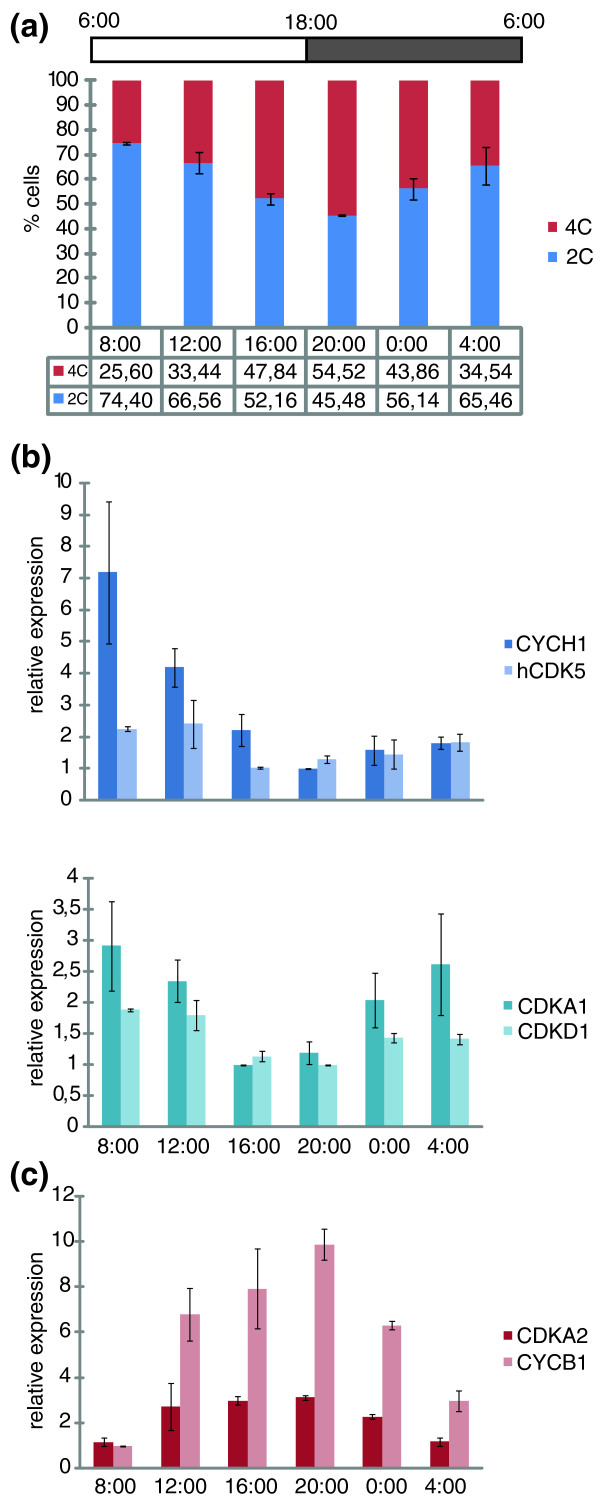
**Validation of cell cycle marker genes**. **(a) **DNA distributions (2C versus 4C) of exponentially growing cells entrained by a LD 12:12 photoperiod during the time series **(b) **Expression profiles of early cell cycle genes (*CYCH1 *and *hCDK5*; peak expression at t = 2 in the synchronization series (Figure 4 and 6)); and *CDKA1 *and *CDKD1 *(peak expression at t = 3 in the synchronization series (Figure 4)). **(c) **Expression profiles of late cell cycle genes (*CDKA2 *and *CYCB1*). Error bars represent standard errors of the mean of two biological replicates.

To validate whether the expression data obtained through the synchronization experiment was applicable in cell cycle-associated studies, we selected diatom cell cycle genes with a defined expression pattern to test their value as cell cycle biomarkers. As a control experiment, we checked the expression of four early (*CYCH1*, *hCDK5*, *CDKA1*, and *CDKD1*) and two late (*CDKA2* and *CYCB1*) cell cycle genes during a 12-h light/12-h dark photoperiod (LD 12:12). Flow cytometry data during this 24-h time course of the grown cultures indicate that the cells show a low degree of 'natural' synchronization of cell division: in the morning, most cells are in the G1 phase, while in the evening, division takes place (Figure 8a). Thus, it was to be expected that genes determined as early and as late cell cycle genes would be induced in the morning and in the evening, respectively. Indeed, expression according to the different cell cycle distributions was found for all selected genes (Figure 8b, c), indicating that they would perform as good cell cycle markers in cell cycle-related studies and that the expression data obtained from the synchronization studies (Figures 4 and 6) could serve as a reliable basis to select appropriate marker genes.

In a real case study, we used these cell cycle biomarkers to investigate whether the cell cycle in *P. tricornutum *would be regulated by an endogenous clock or a so-called circadian oscillator. Circadian regulation of cell division is well known to occur in eukaryotes and is particularly well-described for unicellular algae [87,88]. Although circadian regulation of light-harvesting protein-encoding genes and pigment synthesis has been reported in diatoms [89,90], we did not find any direct evidence that circadian regulation of the cell cycle exists in *P. tricornutum*. Comparison of cell cycle progression and cell cycle biomarker expression in cells under normal LD 12:12 or free-running LL 12:12 light conditions indicate that neither the cell cycle itself nor mRNA accumulation of the main core cell cycle genes depends on a circadian oscillator (Additional files 5 and 6). These findings stress even more the importance of the development and use of efficient signalling networks that link environmental cues to cell growth in diatoms.

## Conclusions

From the annotation and expression analyses, we conclude that the diatom cell cycle machinery shares common features with cell cycle regulatory systems present in other eukaryotes, including a PSTAIRE-containing CDK, conserved cyclin classes of types A, B, and D, and a MYT1 kinase. In addition, members of the retinoblastoma pathway for G1-S regulation involving the retinoblastoma protein and E2F/DP transcription factors [91-93] were also found in *P. tricornutum *(unpublished data). Components that were expected to be found in diatoms but could not be identified include a CDC25 phosphatase and CKIs. Possibly the function of the CDC25 phosphatase might be taken over by *CDKA2*, given its expression time and sequence similarity with B-type CDKs [48], whereas the lack of CKI identification by sequence similarity searches might be due to high sequence divergence [52].

Most interestingly, we found a major expansion of the cyclin gene family in diatoms and discovered a new cyclin class, the diatom-specific cyclins. The latter are most probably involved in signal integration to the cell cycle because transcript levels of *dsCYC5*, *dsCYC7*, and *dsCYC10 *depended on phosphate (this study), and *dsCYC9 *was reported to be induced upon silica availability [81]. Besides their role in nutrient sensing, we hypothesize that transcription of some *dsCYC *genes might also be light-modulated, as illustrated by the high *dsCYC2 *mRNA levels in dark-acclimated cells that drastically dropped after 1 h of light exposure (Figure 6b). In addition, this gene was recently found to be modulated upon blue light treatment [18]. The responsiveness of other *dsCYC *genes to different light conditions is currently under investigation.

The complete set of major diatom key cell cycle regulators identified in this study could serve as a set of marker genes for monitoring diatom growth both in the laboratory and in the field. As cell cycle-regulated transcription cannot be assumed to depict a cell cycle-regulatory role for a gene, the predicted functions of the individual diatom cell cycle genes await further experimental confirmation by molecular and biochemical studies, although they already provide first insights into the manner in which diatoms control their cell division. Therefore, this dataset will form a starting point for future experiments aimed at exploring and manipulating the diatom cell cycle.

## Materials and methods

### Culture conditions

*P. tricornutum *(Pt1 8.6; accession numbers CCAP 1055/1 and CCMP2561) [29] was grown in F/2 medium without silica (F/2-Si) [94], made with filtered and autoclaved sea water collected from the North Sea (Belgium). Cultures were cultivated at 18 to 20°C in a 12-h light/12-h dark regime (50 to 100 μmol·photons·m^-2^·s^-1^) and shaken at 100 rpm. Under these conditions, the average generation time of *P. tricornutum *was calculated to be 0.93 ± 0.07 days (Additional file 3).

### Family-wise annotation of the diatom cell cycle genes

In a first step, known plant and animal cell cycle genes were selected to construct a reference cell cycle dataset. The members of every cell cycle family were used to build family-specific HMMER profiles [95]. With these profiles, the predicted *P. tricornutum *and *T. pseudonana *proteomes were screened for the presence of core cell cycle families. Missing gene families were also screened against the raw genome sequence (using tBLASTN) to account for annotation errors (that is, missing genes). For each family, the putative *P. tricornutum *homologs found were validated by comparing them with the reference family members in a multiple alignment.

### Phylogenetic analysis

Multiple alignments generated with MUSCLE [96] were manually improved with BioEdit [97]. To define subclasses within the gene families, phylogenetic trees were built that included the reference cell cycle genes from plants and animals. Both TREECON [98] and PHYLIP [99] were used to construct the neighbor-joining trees based on Poisson-corrected distances. To test the significance of the nodes, bootstrap analysis was applied using 1,000 replicates for all trees, except for the cyclin tree (500 replicates).

### Synchronization of the cell cycle in *P. tricornutum*

*P. tricornutum *cells were arrested in the G1 phase by prolonged darkness (20 h). After release of the cells from this G1 checkpoint by reillumination, samples for cell cycle analysis and real-time qPCR were collected during 12 h at hourly intervals, starting at reillumination (t = 0). To prevent cells from entering a second cell cycle, nocodazole (2.5 mg/l; Sigma-Aldrich, St. Louis, Missouri, USA) was added to the cultures at t = 0. Synchronization was validated by flow cytometric analysis on a Partec CyFlow ML platform (with data acquisition software Flomax; Partec GmbH, Münster, Germany) on cells fixed with 70% ethanol, washed three times with 1× phosphate buffered saline and stained with 4',6-diamidino-2-phenylindole (final concentration of 1 ng/ml). For each sample, 10,000 cells were processed. Flow cytograms were analyzed with Multicycle AV for Windows (Phoenix Flow Systems, San Diego, California, USA) software to determine relative representations of the different cell cycle stages in the samples.

### Nutrient starvation/repletion experiment

Exponentially growing cells (under constant light, 50 μmol·photons·m^-2^·s^-1^) were collected by centrifugation 3 days after medium replenishment, and washed twice with natural seawater (North Sea, Belgium) to starve the cells. After 24 h starvation, the culture was subdivided into six subcultures and supplied with only nitrate (8.82 × 10^-4 ^M NaNO_3_; N), phosphate (3.62 × 10^-5 ^M NaH_2_PO_4 _H_2_O; P), iron (1.17 × 10^-5 ^M FeCl_3 _6H_2_O; Fe), trace metals (3.93 × 10^-8 ^M CuSO_4 _5H_2_O, 2.60 × 10^-8 ^M Na_2_MoO_4 _2H_2_O, 7.65 × 10^-8 ^M ZnSO_4 _7H_2_O, 4.20 × 10^-8 ^CoCl_2 _6H_2_O and 9.10 × 10^-7 ^M MnCl_2 _4H_2_O; trace), the combination of all nutrients (concentrations as mentioned above; F/2), or no nutrients (no repletion). Samples were taken for real-time qPCR after 3 hours of incubation. Cell density and growth rate were monitored during 3 days after repletion using a Bürker counting chamber to assess the degree of starvation in the different subcultures. For each sample, the average cell density was counted from nine large squares (0.1 mm^3^) and growth rate was calculated from semi-log linear regression of the cell numbers plotted against time.

To exclude cell cycle effects upon nitrate repletion, the experiment was repeated with cells grown in a LD 12:12 photoperiod. Three days after medium replenishment, the cells were washed twice with natural seawater (North Sea, Belgium) to starve the cells and illuminated for 12 h. The cells were then incubated in the dark for 24 h and no nutrients and nitrate were supplied in the dark as mentioned above. Samples were taken for real-time qPCR after 3 hours of incubation in the dark.

### Real-time qPCR

For RNA extraction, 5 × 10^7 ^cells were collected at each time point, fast frozen in liquid nitrogen, and stored at -70°C. To lyse the cells and extract RNA, TriReagent (Molecular Research Center, Inc., Cincinnati, Ohio, USA) was used initially. After addition of chloroform, RNA was purified from the aqueous phase by RNeasy purification, according to the manufacturer's instructions (RNeasy MinElute Cleanup kit; Qiagen, Hilden, Germany). Contaminating genomic DNA was removed by DNaseI (GE Healthcare, Little Chalfont, United Kingdom) treatment. RNA concentration and purity were assessed by spectrophotometry (NanoDrop ND-1000, Wilmington, Delaware, USA). Total RNA was reverse transcribed with Superscript II reverse transcriptase (Invitrogen, Carlsbad, California, USA) in a total volume of 40 μl with oligo(dT) primers. Finally, 1.25 ng (synchronization experiment and control experiment) or 10 ng (nutrient starvation/repletion experiment and circadian experiment) of cDNA was used as template for each qPCR reaction.

Samples in triplicate were amplified on the Lightcycler 480 platform with the Lightcycler 480 SYBR Green I Master mix (Roche Diagnostics, Brussels, Belgium) in the presence of 0.5 μM gene-specific primers (Additional file 1). The cycling conditions were 10 minutes polymerase activation at 95°C and 45 cycles at 95°C for 10 s, 58°C for 15 s, and 72°C for 15 s. Amplicon dissociation curves were recorded after cycle 45 by heating from 65°C to 95°C. In qBase [100], data were analyzed using the ΔC_t _relative quantification method with the stably expressed histone H4 as a normalization gene (Additional file 7) [101]. Expression profiles of the synchronized cell cycle series were mean relative expression from three independent sample series. After normalization, the mean profiles were clustered using hierarchical average linkage clustering (analysis software TIGR MultiExperiment Viewer 3D (TMEV3D)).

### Image acquisition

Confocal images were obtained with a scanning confocal microscope 100 M (Zeiss, Jena, Germany) equipped with the software package LSM510 version 3.2 (Zeiss, Jena, Germany) and a C-Apochromat 63× (1.2 NA) water-corrected objective. Chlorophyll autofluorescence was excited with HeNe illumination (543 nm).

### Accession numbers

Sequence data from this article can be accessed through the Joint Genome Institute (JGI) portal [102]. Accession numbers of the cell cycle genes are listed in Additional file 1.

## Abbreviations

CAK: CDK-activating kinase; CDK: cyclin-dependent kinase; CKI: CDK inhibitor; CKS: CDK subunit; CYC: cyclin; D: dark; dsCYC: diatom-specific cyclin; hCDK: hypothetical CDK; L: light; qPCR: quantitative polymerase chain reaction.

## Authors' contributions

MJJH performed the synchronization and expression experiments, analyzed the data and wrote the manuscript; CM was involved in the genome-wide annotation of cell cycle genes in *P. tricornutum *and *T. pseudonana *and helped write the manuscript; KV and ER were involved in the genome-wide annotation of diatom cell cycle genes. MJJH, CM, KV, JG, ER, MH, CB, DI, YVDP, LDV and WV helped to conceive and design the study, and read and approved the manuscript.

## Supplementary Material

Additional file 1 **Cell cycle genes in *P. tricornutum***. An Excel spreadsheet providing an overview of the annotated cell cycle genes in *P. tricornutum*. Click here for file

Additional file 2 **Cell cycle progression in nocodazole-treated versus untreated cells**. A PDF figure file showing cell cycle progression in nocodazole-treated versus untreated cells. (a) Flow histograms plotting DNA content against cell number (left) and histograms indicating the ploidy distribution (2C versus 4C; right) during a 12-h time course of synchronized cells in the absence of nocodazole. At the later time points (t = 10 to 12), the level of synchrony decreased, indicated by the ploidy level equilibrium reached at these time points, probably resulting from cells entering the next cell cycle round, while other cells still have to pass through M phase. (b) Flow histograms plotting DNA content against cell number (left) and histograms indicating the ploidy distribution (2C versus 4C; right) during a 12-h time course of synchronized cells in the presence of nocodazole. At the later time points, an increasing enrichment of 4C cells can be observed because of a blockage of the cells at metaphase. Asterisk marks the apparently lower proportion of 2C cells after a 20-h dark treatment in the control series than in the nocodazole series, resulting from an acquisition artefact during flow cytometry, indicated by the increased peak broadness in the respective flow histogram. Click here for file

Additional file 3 **Growth curves of *P. tricornutum *cells under standard conditions**. A PDF figure file showing growth curves of *P. tricornutum *cells under standard conditions (18°C, LD 12:12, 50 to 100 μmol·photons·m^-2^·s^-1^). Error bars represent standard deviations. Click here for file

Additional file 4 **Phylogenetic tree of WEE1/MYT1/MIK1 family**. A PDF figure file showing a Phylogenetic tree of WEE1/MYT1/MIK1 family. Neighbor-joining tree (PHYLIP, 1,000 replicates) of WEE1/MYT1/MIK1 family. The *P. tricornutum *sequence is shown in bold. Abbreviations: Arath, *Arabidopsis thaliana*; Drome, *Drosophila melanogaster*; Homsa, *Homo sapiens*; Musmu, *Mus musculus*; Orysa, *Oryza sativa*; Phatr, *Phaeodactylum tricornutum*; Schpo, *Schizosaccharomyces pombe*; Thaps, *Thalassiosira pseudonana*. Click here for file

Additional file 5 **Cell cycle versus circadian control**. A PDF figure file showing cell cycle versus circadian control. Exponentially growing cultures entrained by a LD 12:12 photoperiod were subdivided in two cultures at the end of the light period 3 days after medium replenishment. Left and right: cells experiencing a normal (darkness; grey bar) and subjective (light; white bar) night, respectively. **(a) **Histograms plotting DNA distributions (2C versus 4C) of the cells during the 24-h time series. **(b) **Expression profiles of early cell cycle genes. **(c) **Expression profiles of late cell cycle genes. Error bars represent standard errors of the mean of two biological replicates. Click here for file

Additional file 6 **Cell cycle versus circadian control**. A PDF figure file showing cell cycle versus circadian control. Flow histograms (DNA content plotted against cell number) of the different sampling points depicted in Additional file 5. Click here for file

Additional file 7 **Normalization gene evaluation**. A PDF figure file showing normalization gene evaluation. **(a) **Real-time qPCR cycle threshold (Ct) values of candidate housekeeping genes during a 12-h (sampling every hour) synchronization time series. **(b) **Variation of Ct values of the candidate housekeeping genes during a 12-h (sampling every hour) synchronization time series. Error bars represent standard deviations. Click here for file
