# Supplementary figures and images for: Genome-wide analysis of the diatom cell cycle unveils a novel type of cyclins involved in environmental signaling
Source: Genome Biol. 2010 Feb 8;11(2):R17. doi: 10.1186/gb-2010-11-2-r17 (PMC2872877; doi:10.1186/gb-2010-11-2-r17)

a

*control*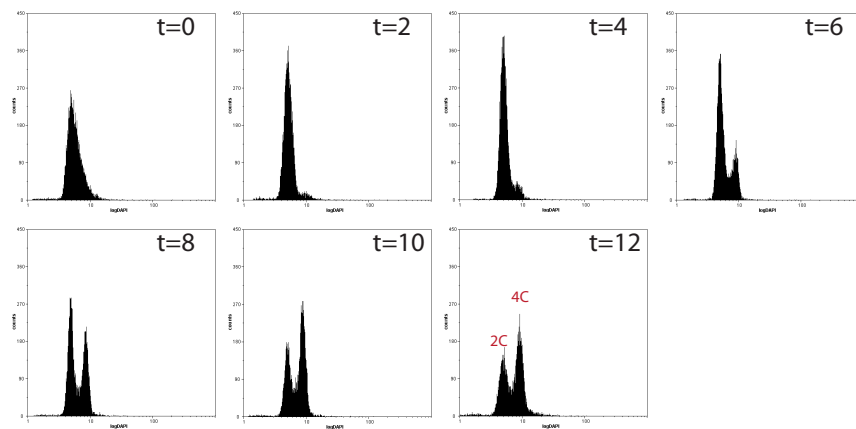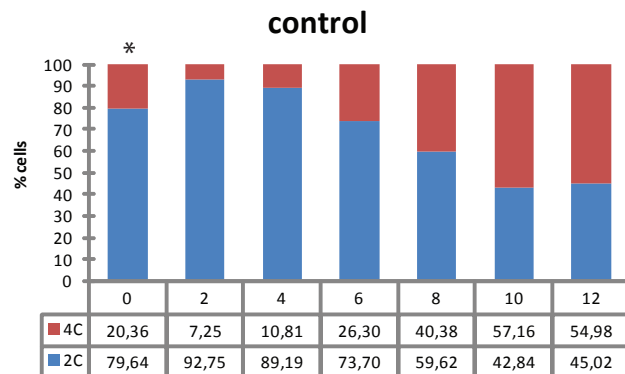

b

*nocodazole treated*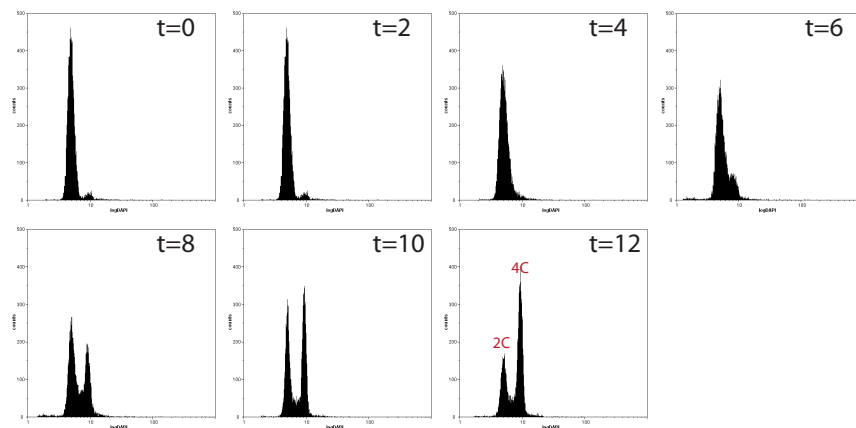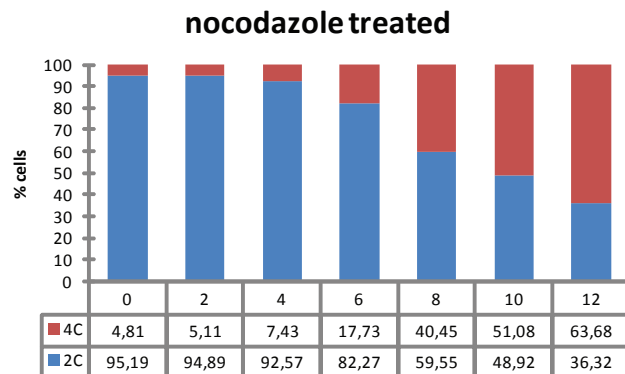

Supplement: Additional file 2 — Cell cycle progression in nocodazole-treated versus untreated cells. A PDF figure file showing cell cycle progression in nocodazole-treated versus untreated cells. (a) Flow histograms plotting DNA content against cell number (left) and histograms indicating the ploidy distribution (2C versus 4C; right) during a 12-h time course of synchronized cells in the absence of nocodazole. At the later time points (t = 10 to 12), the level of synchrony decreased, indicated by the ploidy level equilibrium reached at these time points, probably resulting from cells entering the next cell cycle round, while other cells still have to pass through M phase. (b) Flow histograms plotting DNA content against cell number (left) and histograms indicating the ploidy distribution (2C versus 4C; right) during a 12-h time course of synchronized cells in the presence of nocodazole. At the later time points, an increasing enrichment of 4C cells can be observed because of a blockage of the cells at metaphase. Asterisk marks the apparently lower proportion of 2C cells after a 20-h dark treatment in the control series than in the nocodazole series, resulting from an acquisition artefact during flow cytometry, indicated by the increased peak broadness in the respective flow histogram. [file gb-2010-11-2-r17-S2.pdf]

**culture1**

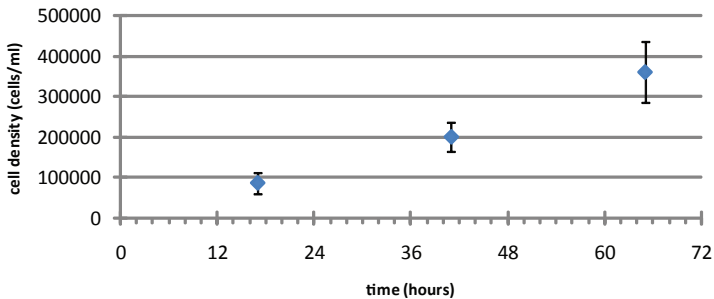

**culture2**

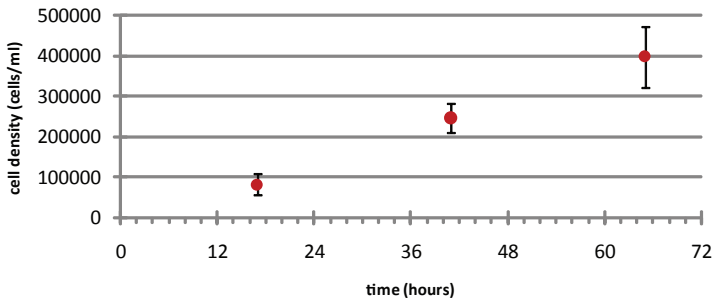

Supplement: Additional file 3 — Growth curves of P. tricornutum cells under standard conditions. A PDF figure file showing growth curves of P. tricornutum cells under standard conditions (18°C, LD 12:12, 50 to 100 μmol·photons·m-2·s-1). Error bars represent standard deviations. [file gb-2010-11-2-r17-S3.pdf]

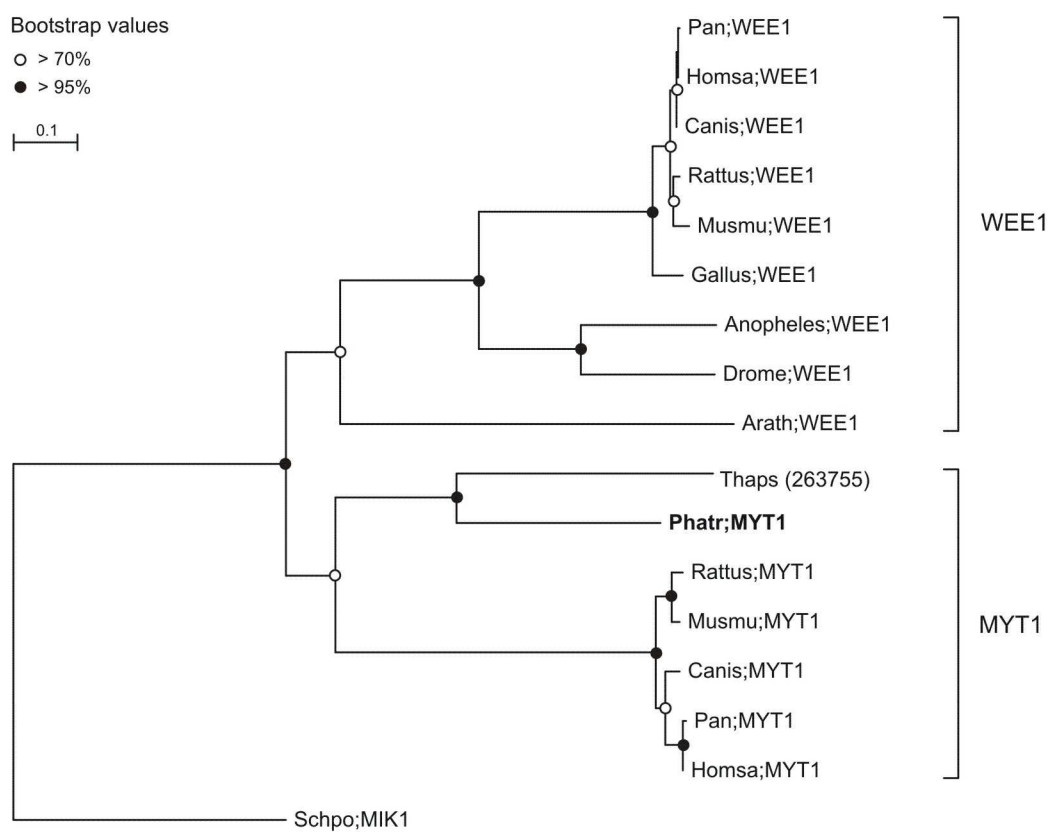

Supplement: Additional file 4 — Phylogenetic tree of WEE1/MYT1/MIK1 family. A PDF figure file showing a Phylogenetic tree of WEE1/MYT1/MIK1 family. Neighbor-joining tree (PHYLIP, 1,000 replicates) of WEE1/MYT1/MIK1 family. The P. tricornutum sequence is shown in bold. Abbreviations: Arath, Arabidopsis thaliana; Drome, Drosophila melanogaster; Homsa, Homo sapiens; Musmu, Mus musculus; Orysa, Oryza sativa; Phatr, Phaeodactylum tricornutum; Schpo, Schizosaccharomyces pombe; Thaps, Thalassiosira pseudonana. [file gb-2010-11-2-r17-S4.pdf]

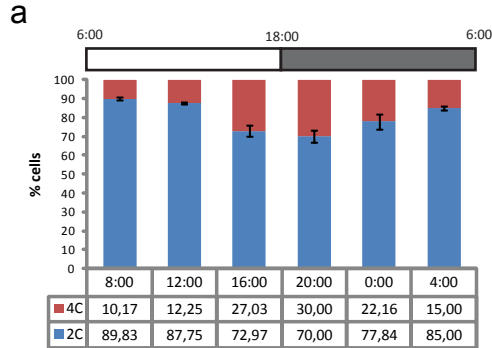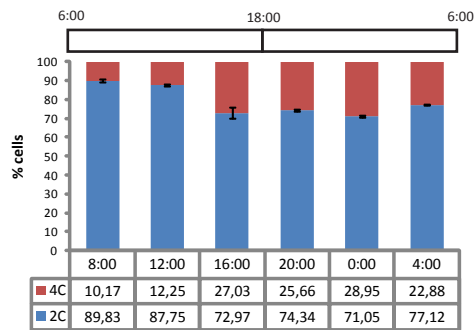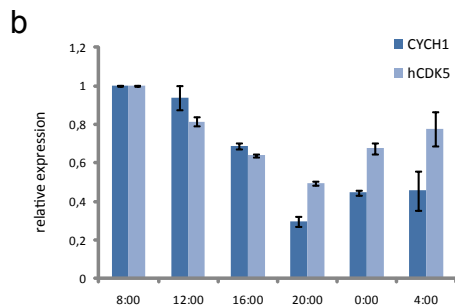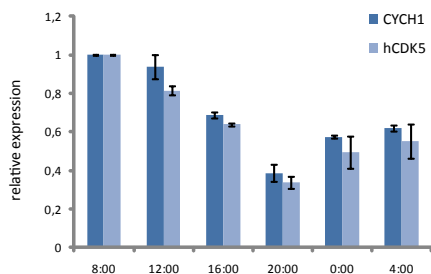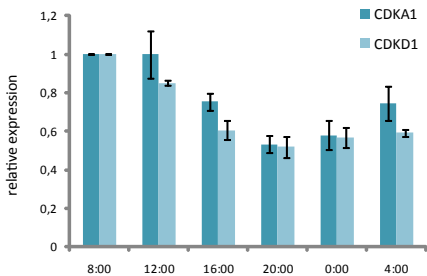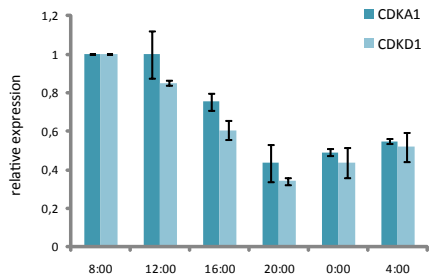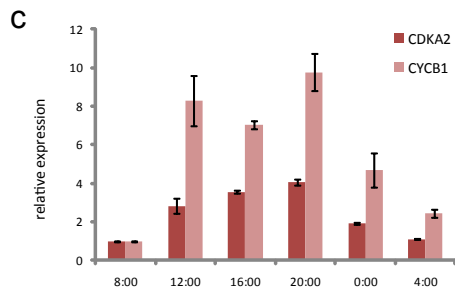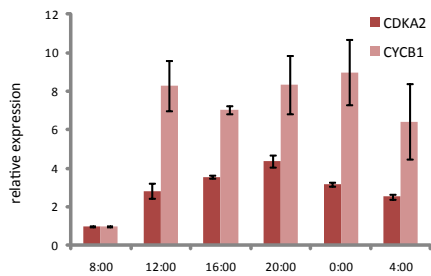

Supplement: Additional file 5 — Cell cycle versus circadian control. A PDF figure file showing cell cycle versus circadian control. Exponentially growing cultures entrained by a LD 12:12 photoperiod were subdivided in two cultures at the end of the light period 3 days after medium replenishment. Left and right: cells experiencing a normal (darkness; grey bar) and subjective (light; white bar) night, respectively. (a) Histograms plotting DNA distributions (2C versus 4C) of the cells during the 24-h time series. (b) Expression profiles of early cell cycle genes. (c) Expression profiles of late cell cycle genes. Error bars represent standard errors of the mean of two biological replicates. [file gb-2010-11-2-r17-S5.pdf]

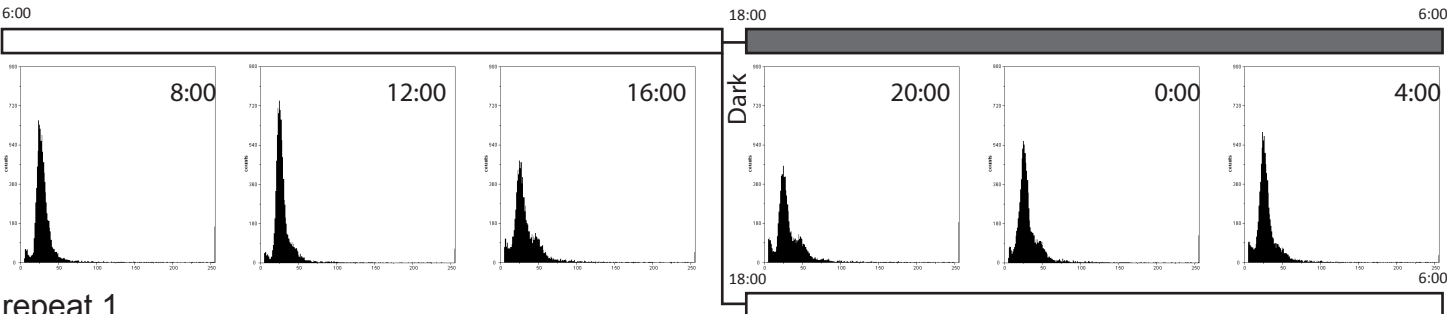

repeat 1

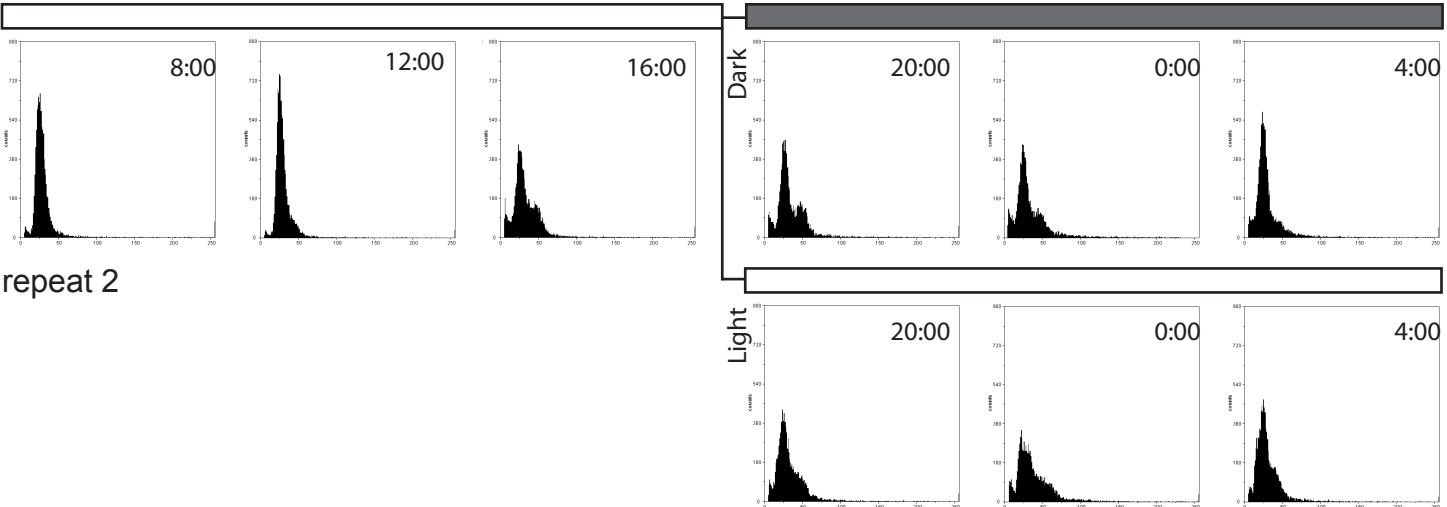

repeat 2

Supplement: Additional file 6 — Cell cycle versus circadian control. A PDF figure file showing cell cycle versus circadian control. Flow histograms (DNA content plotted against cell number) of the different sampling points depicted in Additional file 5. [file gb-2010-11-2-r17-S6.pdf]

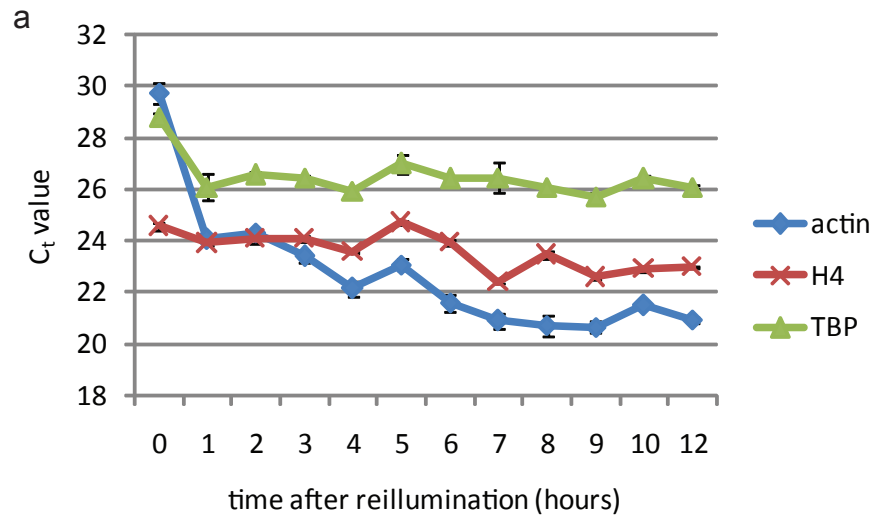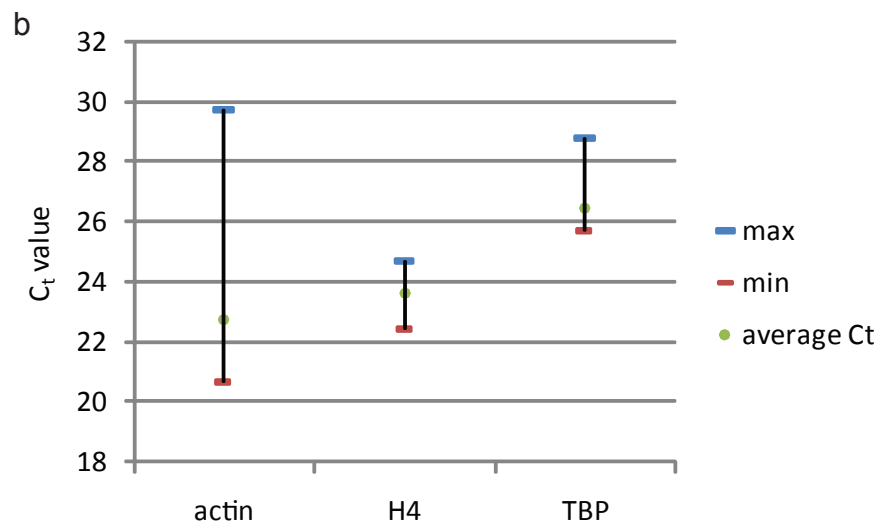

Supplement: Additional file 7 — Normalization gene evaluation. A PDF figure file showing normalization gene evaluation. (a) Real-time qPCR cycle threshold (Ct) values of candidate housekeeping genes during a 12-h (sampling every hour) synchronization time series. (b) Variation of Ct values of the candidate housekeeping genes during a 12-h (sampling every hour) synchronization time series. Error bars represent standard deviations. [file gb-2010-11-2-r17-S7.pdf]
